# Analysis of oncogenic activities of protein kinase D1 in head and neck squamous cell carcinoma

**DOI:** 10.1186/s12885-018-4965-6

**Published:** 2018-11-12

**Authors:** Liyong Zhang, Zhihong Li, Yehai Liu, Shuping Xu, Manuj Tandon, Brittany Appelboom, Courtney R. LaValle, Simion I. Chiosea, Lin Wang, Malabika Sen, Vivian W. Y. Lui, Jennifer R. Grandis, Q. Jane Wang

**Affiliations:** 1 Department of Pharmacology and Chemical Biology, University of Pittsburgh School of Medicine, E1354 BST, Pittsburgh, PA 15261 USA; 2 Department of Otolaryngology, University of Pittsburgh, Pittsburgh, PA 15261 USA; 3 Department of Pathology, University of Pittsburgh, Pittsburgh, PA 15261 USA; 4 Department of Biochemistry, China Three Gorges University, Yichang, Hubei Province People’s Republic of China 443002; 5 Department of Otolaryngology, Head and Neck Surgery, First Affiliated Hospital of Anhui Medical University, Hefei, Anhui Province People’s Republic of China 230022; 6 Present address: Otolaryngology/Head and Neck Surgery, University of California, San Francisco, CA 94115 USA; 7 School of Biomedical Sciences, Faculty of Medicine, Chinese University of Hong Kong, Hong Kong, Hong Kong SAR

**Keywords:** PKD1, GRP/bombesin, Head and neck cancer, Tumor growth, Proliferation

## Abstract

**Background:**

Head and neck squamous cell carcinoma (HNSCC) is the sixth leading cause of cancer death in the US. The protein kinase D (PKD) family has emerged as a promising target for cancer therapy with PKD1 being most intensively studied; however, its role in HNSCC has not been investigated.

**Methods:**

The expression of PKD was evaluated in human HNSCC by quantitative RT-PCR, Western blot and immunohistochemistry. Cell proliferation, wound healing, and matrigel invasion assays were performed upon siRNA-mediated knockdown of PKD1 in HNSCC cells, and subcutaneous xenograft mouse model was established by implantation of the stable doxycycline (Dox)-inducible PKD1 expression cell lines for analysis of tumorigenic activity in vivo.

**Results:**

PKD1 was frequently downregulated in HNSCC cell lines at both transcript and protein levels. In human HNSCC tissues, PKD1 was significantly down-regulated in localized tumors and metastases, and in patient-paired tumor tissues as compared to their normal counterparts, which was in part due to epigenetic modification of the *PRKD1* gene. The function of PKD1 in HNSCC was analyzed using stable doxycycline-inducible cell lines that express native or constitutive-active PKD1. Upon induction, the rate of proliferation, survival, migration and invasion of HNSCC cells did not differ significantly between the control and PKD1 overexpressing cells in the basal state, and depletion of endogenous PKD1 did not impact the proliferation of HNSCC cells. However, the median growth rate of the subcutaneous HNSCC tumor xenografts over time was elevated with PKD1 induction, and the final tumor weight was significantly increased in Dox-induced vs. the non-induced tumors. Moreover, induced expression of PKD1 promoted bombesin-induced cell proliferation of HNSCC and resulted in sustained ERK1/2 activation in response to gastrin-releasing peptide or bombesin stimulation, suggesting that PKD1 potentiates GRP/bombesin-induced mitogenic response through the activation of ERK1/2 in HSNCC cells.

**Conclusions:**

Our study has identified PKD1 as a frequently downregulated gene in HNSCC, and functionally, under certain cellular context, may play a role in GRP/bombesin-induced oncogenesis in HNSCC.

**Electronic supplementary material:**

The online version of this article (10.1186/s12885-018-4965-6) contains supplementary material, which is available to authorized users.

## Background

The protein kinase D (PKD) family of serine/threonine kinases belongs to the Ca^2+^/calmodulin-dependent protein kinase (CaMK) superfamily. The three isoforms (PKD1, 2, 3) of PKD are widely distributed in a variety of tissues and show high sequence homology [1–3]. Several conserved structure domains are present in PKD, including a diacylglycerol-binding C1 domain and a PH domain that exerts an autoinhibitory function to the kinase activity. PKD can be activated by PKC-mediated trans-phosphorylation of two conserved serine residues (Serine 738/742 in human PKD1) in the activation loop of PKD [4]. Sustained PKD activation can be maintained via PKC-independent autophosphorylation events [5]. Through the DAG/PKC/PKD axis, PKD plays an important role in propagating signals from G protein-coupled receptors (GPCRs) and growth factor receptors at the cell surface.

PKD has been implicated in multiple cancers. Altered PKD expression and activity have been demonstrated in prostate, breast, pancreas, skin, and gastric cancers [1, 6]. PKD1 is the most intensely studied PKD isoform to date. In certain cancers including pancreatic and skin cancer, higher PKD1 expression and activity were detected in tumors as compared to normal tissues and increased PKD1 expression was associated with hyper-proliferative phenotype and increased tumor aggressiveness [6, 7]. Interestingly, in other cancer types including breast, gastric and prostate cancer, PKD1 was found to be downregulated in primary tumors or metastases [8–11]. Reduced PKD1 expression was associated with increased tumor invasiveness and its overexpression in prostate cancer cells was shown to inhibit tumor cell proliferation [10, 12]. Thus, the functional relevance of PKD1 to tumor initiation and progression remains to be determined.

Head and neck squamous cell carcinoma (HNSCC) is one of the most common type of human cancers. The annual incidence is more than 500,000 cases worldwide. There are more than 40,000 new cases of HNSCC reported in the United States, and nearly 12,000 will die from the disease [13]. The origin of HNSCC involves multiple organs, including the oral cavity, pharynx, and larynx. HNSCC at early-stage (Stage I and II) can be curatively treated with surgery or radiotherapy. However, advanced HNSCC (stage III and IV) remains an aggressive disease that is associated with high morbidity and mortality. The 3-year survival rate for patients with advanced disease under standard therapy is only 30–50%, and a large number of these patients (nearly 40% to 60%) subsequently develop locoregional recurrences or distant metastases [13–15]. Despite the advances in treatment strategies, the survival rates for patients with advanced HNSCC have not improved significantly, underscoring an urgent need to better understand the molecular mechanisms underlying the pathogenesis of HNSCC. The role of PKD in HNSCC has not been fully investigated. The current study was undertaken to evaluate the expression of PKD1 in HNSCC tumor specimens and cell lines to gain insights into its clinical significance. The study also sought to investigate the functional implication of PKD1 in HNSCC by systematically determining its cancer-associated biological properties in HNSCC cells in vitro and in vivo and to assess the potential value of targeting PKD1 for cancer therapy.

## Methods

### Materials

Doxycylcline hyclate, 5-Aza-2′-deoxycytidine (5-aza-dC), gastrin-releasing peptide, and DMSO were obtained from Sigma-Aldrich (St. Louis, MO). The histone deacetylase HDAC inhibitor suberoylanilide hydroxamic acid (SAHA) was purchased from Cayman Chemical (Ann Arbor, MI). Bombesin was obtained from Fisher Scientific (Pittsburgh, PA).

### Immunohistochemistry (IHC)

The normal and malignant human head and neck tissue sections were obtained from US Biomax (Rockville, MD, www.biomax.us) and Pantomics (Richmond, CA, www.pantomics.com). The patient-paired tumor specimens were obtained from Dr. Yehai Liu at the Department of Otolaryngology, Head and Neck Surgery, First Affiliated Hospital of Anhui Medical University, Hefei, Anhui, China. A written informed consent from the donor or the next of kin was obtained for the use of these samples in research. The tissues were collected following a named medically prescribed procedure during standard treatment without authors’ involvement. The fully randomized and de-identified samples were provided to the authors. These samples are exempt from the requirement of IRB approval (Exempt Category 4) since they are de-identified and publicly available. For IHC staining, the tissue sections were dewaxed with xylene and rehydrated through gradient ethanol into water. For antigen retrieval, sections were heated in citrate buffer (pH 6.0) for 10 min at 95 °C. The sections were then digested with 0.05% trypsin for 10 min at 37 °C. Endogenous peroxidase activity was quenched with 0.3% H_2_O_2_ in methanol for 30 min at room temperature. After washing with PBS, slides were pre-blocked with 10% normal goat non-immune serum at 37 °C for 30 min. Sections were incubated with primary antibody targeting PKD1 (1:150) at 4 °C overnight, washed with PBS, and incubated with biotinylated secondary antibody at a 1:200 dilution for 30 min. The sections were then developed by incubating first in Vectastain ABC reagent (Vector Laboratories, Inc., Burlington, CA) and then with 3,3′-diaminobenzidine (Sigma-Aldrich). Slides were counterstained with hematoxylin, dehydrated, and mounted on coverslips. Negative controls were obtained by omitting the primary antibody. The staining was scored independently by two experienced researchers according to the number and intensity of immunopositive cells in a blinded fashion. The percentage of positive tumor cells was determined semi-quantitatively by assessing the entire tumor section and each sample was scored based on the following criteria: 0 (0–4%), 1 (5–24%), 2 (25–49%), 3 (50–74%), or 4 (75–100%). The intensity of immunostaining was categorized as 0 (negative), 1+ (weak), 2+ (moderate) or 3+ (strong). A final immunoreactive score between 0 and 12 was calculated by multiplying the two scores. These procedures were adapted from previously published studies [16–18].

### Cell lines and culture conditions

HNSCC cell lines UMSCC-1, UMSCC-10A, UMSCC22B, Cal33, UPCI 4B, UPCI 15B, 1483 and 686LN were obtained from Dr. Jennifer R. grandis (University of California, San Francisco, CA) as described previously [19]. Het-1A (CRL-2692), a human esophageal squamous epithelial cell line, was obtained from ATCC (Manassas, VA) and cultured in Airway Epithelial Cell Basal Growth Medium with supplement mix (Promo Cell, Heidelberg, Germany). UMSCC-1, Cal33, UMSCC-10A, UMSCC22B, UPCI 4B, UPCI 15B and 1483 cells were maintained in Dulbecco’s Modified Eagle Medium (DMEM) (Fisher Scientific, Pittsburgh, PA) supplemented with 10% fetal bovine serum (FBS) (Invitrogen, Carlsbad, CA), penicillin (100 U/ml)/streptomycin (100 μg/ml) at 37 °C in a humidified atmosphere of 5% CO_2_. OSC19 cells were cultured in Eagle’s Minimum Essential Medium (EMEM) (Fisher) plus 10% FBS and Non-Essential Amino Acid (Fisher). 686LN cells were maintained in Ham’s F-12 medium (Fisher) containing 10% FBS, 100 units/ml penicillin, and 100 μg/ml streptomycin. All cell lines were authenticated by the Research Animal Diagnostic Laboratory by species-specific PCR testing within 6 months of use.

### Development of stable doxycycline (Dox)-inducible PKD1 expression cell lines

The doxycycline-inducible PKD1 expression cell lines were developed using the Tet-On 3G System from Clontech (Mountain View, CA). Briefly, wild-type or constitutive-active (CA) PKD1 gene was sub-cloned into a pTRE3G-based expression vector to generate the pTRE3G-PKD1 or pTRE3G-PKD1-CA plasmid. Meanwhile, UMSCC-1 and 686LN cells were transfected with the pCMV-Tet3G plasmid and selected with G418 to generate stable Tet-On 3G cell lines that constitutively expressed the Tet-On 3G transactivator protein. The stable Tet-On 3G cell lines were then transfected with the pTRE3G-PKD1 or pTRE3G-PKD1-CA plasmid, along with a linear selection marker for puromycin. The cells were then selected with puromycin to generate double-stable cell lines that expressed PKD1 or PKD1-CA in response to Dox treatment. The stable clones were isolated and the induction of PKD1 was confirmed by Western blotting analysis. Optimal Dox induction condition was determined in a time- and concentration-response experiment, and 500 and 50 ng/ml for 48 h were selected as the optimal induction conditions for UMSCC-1 and 686LN cells, respectively.

### Western blotting

Western blotting analysis was conducted as previously described [20]. Primary antibodies used for Western blotting were from the following sources: PKD1, p-S916-PKD1, p-S744/748-PKD1 antibodies were obtained from Cell Signaling Technology (Danvers, MA); p-Ser742-PKD1 antibody was from Invitrogen (Carlsbad, CA); PKD2 antibody was from Abcam (Cambridge, MA); glyceraldehyde-3-phosphate dehydrogenase (GAPDH) antibody was obtained from Enzo (Farmingdale, NY); tubulin antibody was from Santa Cruz Biotechnology (Santa Cruz, CA).

### Quantitative real-time RT-PCR

Total RNA was prepared using RNeasy Mini Kit according to manufacturer’s instructions (Qiagen, Valencia, CA). Reverse-transcription and real-time quantitative PCR were performed as previously described [21]. Sequences of the primer pairs used were as follows: PKD1, CGCACATCATCTGCTGAACT (forward) and CTTTCGGTGCACAACGTTTA (reverse); PKD2, GGGCAGTTTGGAGTG GTCTA (forward) and ACCAGGATCTGGGTGATGAG (reverse); PKD3, CATGTCCACCAGGAACCAAG (forward) and GACGGGTGTAAGAGTGAACAGC (reverse); GAPDH, GCAAATTCCATGGCACCGT (forward) and TCGCCCCACTTGATTTTGG (reverse). The PCR protocol included 1 cycle at 95 °C for 30 s, then 40 cycles of a 95 °C for 5 s step followed by 5 s at either 62 °C or 65 °C, depending on the optimal annealing temperature for each primer set. Melt curves were conducted to assure specificity of the primer sets as well as absence of primer-dimers.

### siRNA-mediated knockdown of PKD1

Transient knockdown of PKD1 was achieved using multiple siRNAs targeting different regions of *PRKD1* gene. Two validated Stealth PKD1 siRNAs (si-PKD1–1 and si-PKD1–2) and a BLOCK-iT PKD1 siRNA (si-PKD1–3: GUCGAGAGAAGAGGUCAAATT) were obtained from Invitrogen. The sequence for the PKD2-targeting siRNA (si-D2–2) is UCAUCACCCAGAUCCUGGUGGCUUU. The HNSCC cell lines were transiently transfected using DharmaFECT Reagent 3 (Dharmacon, Lafayette, CO) according to the manufacturer’s instructions. Cells were harvested after two days and the levels of PKD1 or PKD2 knockdown were assessed by Western blotting analysis.

### Cell proliferation assay, wound healing assay, and Matrigel invasion assay

Cell proliferation was determined for UMSCC-1 cells transfected with PKD1 siRNAs and stable PKD1-inducible UMSCC-1 and 686LN clones by counting cell numbers for seven consecutive days as previously reported [22]. Growth media with or without Dox was refreshed every 2 days. Cell migration was measured by wound healing assay as previously described [23]. The average % wound healing was determined based on 4 measurements of the wound area. Cell invasion was determined by Matrigel invasion assay as described before [24]. For stable inducible clones, cells were incubated with Dox for 48 h prior to seeding in BD Matrigel invasion or control inserts (BD Biosciences, San Jose, CA). Dox was added to the top and bottom chambers of control and invasion inserts.

### Subcutaneous xenograft mouse model

Six-week old female athymic (NCr) nu/nu mice (NCI-Frederick Cancer Research Facility, Frederick, MD) were randomized into two groups (10 mice/group) for injection of control cells expressing empty vector (control group) or cells expressing stable inducible PKD1 (PKD1 group). The cells (4 × 10^6^ cells) mixed 1:1 with Matrigel (BD Biosciences) were injected subcutaneously into both flanks of mice. Once tumors were palpable, mice in each group were divided to receive either drinking water or Dox-containing driving water (1 mg/mL). Water was changed every 2 days. Tumor size and mouse weight were monitored 2–3 times per week. Tumor size was measured as described [21]. The experiment was terminated after 25 days and tumors were dissected for subsequent analysis. All animal studies were conducted in accordance with IACUC guidelines at the University of Pittsburgh.

### Statistical analysis

All statistical analyses were performed using GraphPad Prism software. The significance between data points from cell proliferation, wound healing, and invasion experiments was assessed by Student’s *t*-test. The Mann-Whitney-Wilcoxon test was used for the tumor xenograft study. A *p*-value of < 0.05 was considered statistically significant.

## Results

### Expression of PKD1 was downregulated in human HNSCC cell lines and tumor specimens

**Fig. 1:**
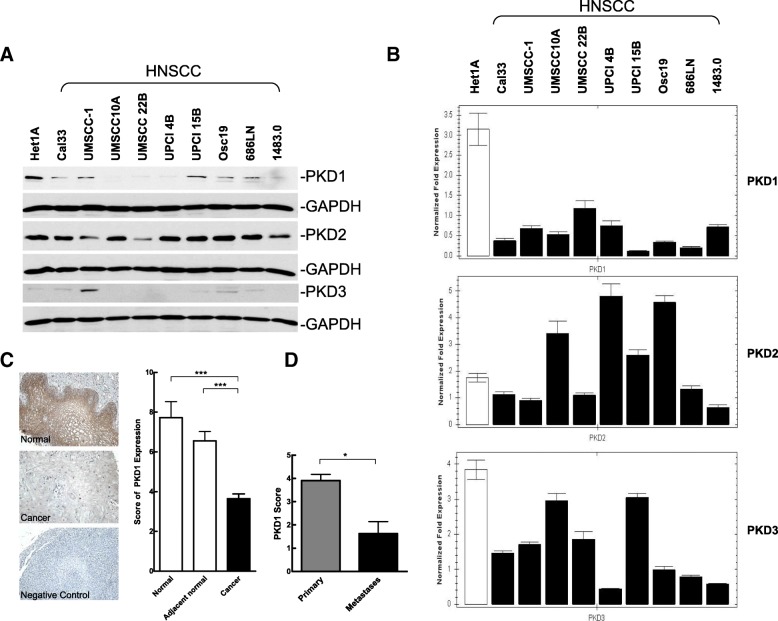
Levels of PKD proteins and transcripts in HNSCC cell lines and analysis of PKD1 expression in normal and malignant human head and neck tissues. **a** PKD expression in normal (Het1A) and malignant head and neck cancer cell lines. Cells were cultured under standard conditions. Cell lysates were subjected to Western blotting for PKD1–3 and GAPDH. The experiment was repeated at least three times and a representative image is shown. **b** PKD mRNA levels in HNSCC cells. PKD transcript levels were determined by quantitative real time RT-PCR analysis, GAPDH was measured as control. Each experiment was repeated three times and representative data are shown. **c** Expression of PKD1 in sporadic head and neck tissue specimens, including 124 cases of head and neck tumor samples and 18 normal or 56 adjacent normal samples. *Left*, representative images of IHC staining of PKD1 in normal and tumor tissue. Negative control, no primary antibody added. Magnification, 200x. *Right*, total score of PKD1 expression. A final PKD1 expression score was calculated by multiplying the scores for intensity and frequency of PKD1 staining in a given tissue specimen. **d** Summary of PKD1 expression in 107 primary tumors and 16 metastases (15 Lymph nodes and 1 liver). Statistics was performed using SPSS. *, *p* < 0.05

The expression of PKD isoforms was evaluated in a panel of nine head and neck cancer cells, including Cal33, UMSCC-1, UMSCC-10A, UMSCC22B, UPCI 4B, UPCI 15B, OSC19, 686LN, and 1483. Het-1A, a normal human esophageal squamous epithelial cell line, was included as a control. As shown in Fig. 1a, PKD1 protein levels were significantly lower in all of the HNSCC cell lines examined as compared to the control Het-1A cells. In contrast, levels of PKD2 protein were similar or slightly higher in HNSCC cell lines than in Het-1A with the exception of three lines showing reduced PKD2 expression (UMSCC-1, UMSCC22B and 1483). PKD3 was minimally expressed in the control and in almost all HNSCC cell lines examined. A similar trend was found in the transcript levels of PKD1 and 2 (Fig. 1b). PKD1 transcript levels were significantly lower in all 9 HNSCC cell lines as compared to that in Het-1A. Additionally, levels of PKD2 were higher in UMSCC-10A, UPCI 4B, UPCI 15B, and OSC19, and lower in Cal33, UMSCC-1, UMSCC22B, 686LN, and 1483 cells, which correlates to the protein levels of PKD2 found in these cell lines (Fig. 1a). Taken together, among members of the PKD family, PKD1 was the only isoform whose protein and transcript levels were persistently downregulated in HNSCC cell lines.

PKD1 expression was further analyzed by immunohistochemistry in a total of 124 sporadic head and neck tumor tissues and 74 normal tissue specimens, among which 56 were normal tissues adjacent to the tumors (sporadic). As shown in Fig. 1c, PKD1 expression in primary HNSCC tissues was significantly lower as compared to the normal and adjacent normal tissues (*p* = 0.0001). Among the tumor specimens, six (5%) showed strong PKD1 expression, ten (8%) moderate, 49 (40%) weak, and 58 (47%) were negative. In contrast, in the normal tissues, 26 (35%) showed strong PKD1 expression, seven (9%) moderate, 27 (36%) weak, and 14 (19%) were negative. There was no significant correlation between PKD1 expression and pathologic grade or depth of primary tumor invasion (T status), neither was there significant association with age and gender (Table 1). In normal squamous mucosa, strong membranous pattern of PKD1 staining as well as diffused or granular cytoplasmic staining of PKD1 were observed, which is in contrast to the weak and diffused cytoplasmic staining of PKD1 in tumor tissues. The stroma surrounding the tumors showed little PKD1 staining. Interestingly, in a small cohort of metastases (15 lymph node metastases and one liver metastasis), a significant reduction of PKD1 expression was observed in distal metastases as compared to the localized primary tumors (*p* = 0.002), suggesting a reverse correlation with tumor metastasis (Fig. 1d).

**Table 1:** Correlations between PKD1 expression and clinicopathological characteristics of human head and neck cancer

	Number	Score of PKD1 expression	*P*
Gender			
Female	18	4.611 ± 0.852	0.116
Male	105	3.514 ± 0.248	
Age			
≤ 35	12	3.083 ± 0.988	0.579 (Group I vs. Group 2)
35–55	79	3.557 ± 0.325	0.629 (Group I vs. Group 3)
≥ 55	36	3.527 ± 0.353	0.958 (Group 2 vs. Group 3)
Depth of invasion			
T1 + T2	45	4.644 ± 0.287	0.063
T3 + T4	41	3.512 ± 0.269	
Clinical stages			
Grade I	24	3.521 ± 0.462	0.253 (Grade I vs. Grade II)
Grade II	19	4.447 ± 0.476	0.726 (Grade I vs. Grade III)
Grade III	43	3.756 ± 0.455	0.341 (Grade II vs. Grade III)

**Fig. 2:**
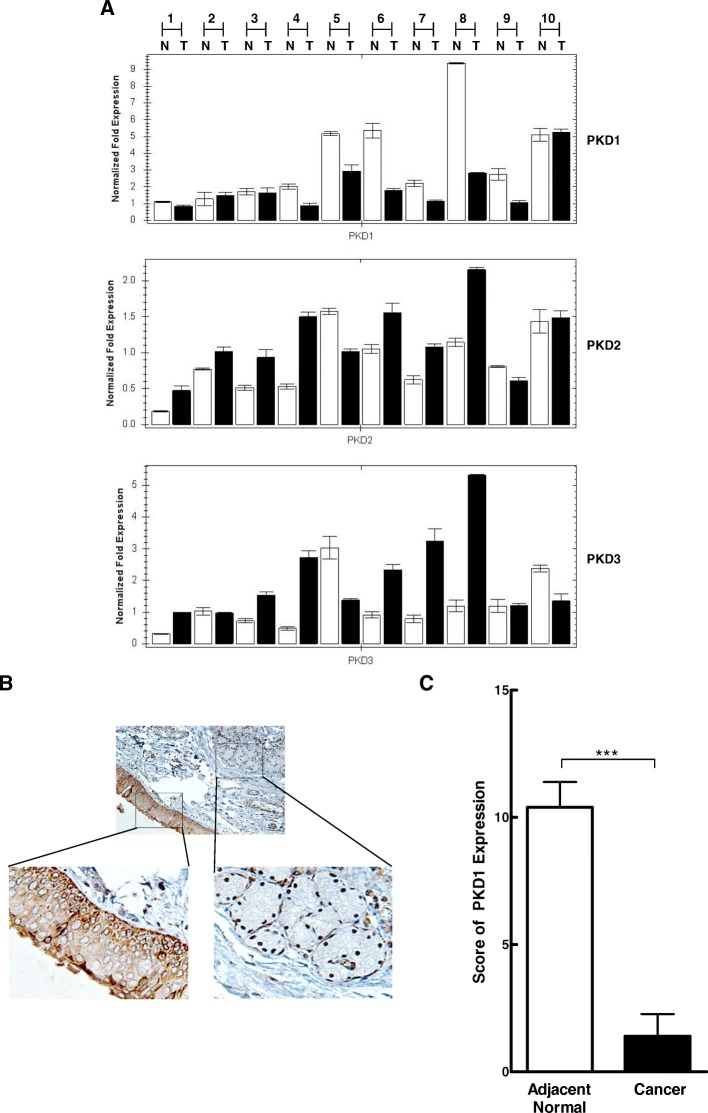
Expression of PKD1 in patient-paired head and neck tissue specimens. **a** mRNAs of patient-paired normal (N) and cancerous (C) head and neck tissue specimens were analyzed for PKD1, 2 and 3 expression by real time RT-PCR. GAPDH was determined as control. **b** IHC staining of PKD1 in patient-paired head and neck tissue specimens (6 pairs of cancer/normal tissues). A representative image of PKD1 staining is shown with a section of tumor and its adjacent normal tissues enlarged. Magnification, 200x. **c** Summary of PKD1 expression in patient-paired head and neck tissue specimens. Statistics was performed using SPSS. *, *p* < 0.05

The pattern of PKD1 expression was further confirmed in a small cohort of patient-paired head and neck normal and tumor tissue samples. As shown in Fig. 2a, quantitative RT-PCR was performed on RNAs obtained from 10 patient-paired normal and tumor tissues of HNSCC. Seven out of the ten (70%) paired samples showed significant downregulation of PKD1 transcript in tumors compared with the normal, and the remaining 3 out of 10 (30%) showed equal levels of PKD1. In contrast, transcript levels of PKD2 and PKD3 showed the opposite trend, i.e. increased PKD2 or PKD3 expression in tumor vs. normal tissue. Specifically, for PKD2, the numbers of patient paired samples that showed increased, reduced or unchanged PKD2 expression in tumor vs. normal tissues were 7, 2 and 1, and for PKD3, the numbers were 6, 2 and 2, respectively. Thus, PKD2 and PKD3 were differentially implicated in HNSCC as compared to PKD1. PKD1 protein expression was further assessed by IHC in a set of 6 patient-paired head and neck normal and tumor tissue samples. PKD1 expressed abundantly in the normal squamous mucosa (enlarged image, left), but little was detected in the adjacent tumor tissues (enlarge image, right) (Fig. 2b). Quantitative analysis indicated a nearly ten-fold reduction in PKD1 expression in the adjacent tumors (*p* < 0.001) (Fig. 2c). Additionally, analysis of the mRNA expression data from the TCGA cohort via cBioPortal demonstrated low mRNA expression in most tumor tissues (Additional file 1: Figure S1A), and PKD1 mRNA was downregulated in 87% HNSCC tumors (459 out of 530 cases with over 2-fold mRNA downregulation), which is consistent with our findings. Taken together, these results indicated that PKD1 expression was frequently reduced in tumors of head and neck cancer patients.

### PKD1 downregulation was independent of DNA methylation or histone acetylation in most head and neck cancer cells

**Fig. 3:**
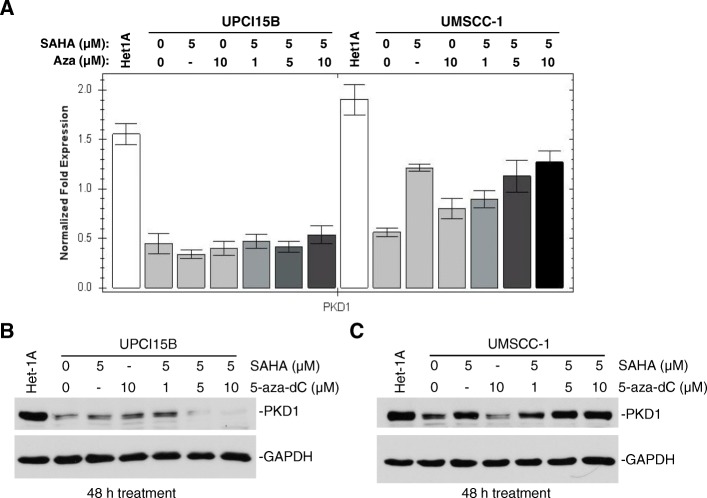
Effects of DNA methyltransferase inhibitor (Aza) and HDAC inhibitor (SAHA) on PKD1 expression. UPCI15B and UMSCC-1 cells were treated with SAHA and 5-aza-dC alone or in combination for 48 h. Cells were harvested for mRNA extraction and Western blotting. Levels of PKD1 transcripts were determined by real time qRT-PCR (**a**). Protein expression was analyzed by immunoblotting for PKD1 (**b** and **c**). GAPDH was used as loading control in both experiments. Representative data from one of three independent experiments are shown

PKD1 gene expression has been shown to be regulated by epigenetic modifications [8, 11] and DNA methyltransferase inhibitors can induced PKD1 expression [8]. To determine if DNA methylation or histone acetylation accounts for the reduced PKD1 gene expression in HNSCC, head and neck cancer cells were treated with DNA methyltransferase inhibitor, 5-aza-2′-deoxycytidine (5-aza-dC), and/or histone deacetylase HDAC inhibitor suberoylanilide hydroxamic acid (SAHA) and trichostatin A (TSA), and levels of PKD1 gene and protein expression was examined. Interestingly, in contrast to previous reports, treatment with SAHA or 5-aza-dC did not significantly alter PKD1 expression at both mRNA and protein levels in four out of five HNSCC cell lines examined with the exception of UMSCC-1 (Fig. 3 and Additional file 1: Figure S2). As shown in Fig. 3a-b, UPCI 15B cells treated with either SAHA or 5-aza-dC did not significantly impact the expression of PKD1 at both mRNA and protein levels. In contrast, UMSCC-1 cells incubated with SAHA (5 μM) for 48 h significantly restored PKD1 expression at both mRNA and protein levels. Although treatment with 5-aza-dC (10 μM) alone had little effect, the combined treatment with increasing concentrations of 5-aza-dC (1, 5, 10 μM) enhanced the effect of SAHA, indicating a potential additive action of the two inhibitors. Data obtained with TSA treatment were similar to those with SAHA in UPCI15B cells (Additional file 1: Figure S2D). Thus, epigenetic modification did not play a major role in the control of PKD1 gene expression in most of the HNSCC cell lines examined, with the exception of UMSCC-1 cells in which histone acetylation appeared to be the major cause of reduced PKD1 gene expression. An analysis of 530 HNSCC tumors from the TCGA via cBioPortal demonstrated low levels of DNA methylation on PRKD1 gene (Additional file 1: Figure S1B). Further analysis indicated 13% cases (67 out of 530 cases) of PKD1 had loss of heterozygosity (LOH), while only three cases (< 1%) of PKD1 showed homozygous deletion. Thus, a combination of genetic and epigenetic alterations contributed to the downregulation of PKD1 expression.

### PKD1 did not significantly alter the proliferation of HNSCC cells

**Fig. 4:**
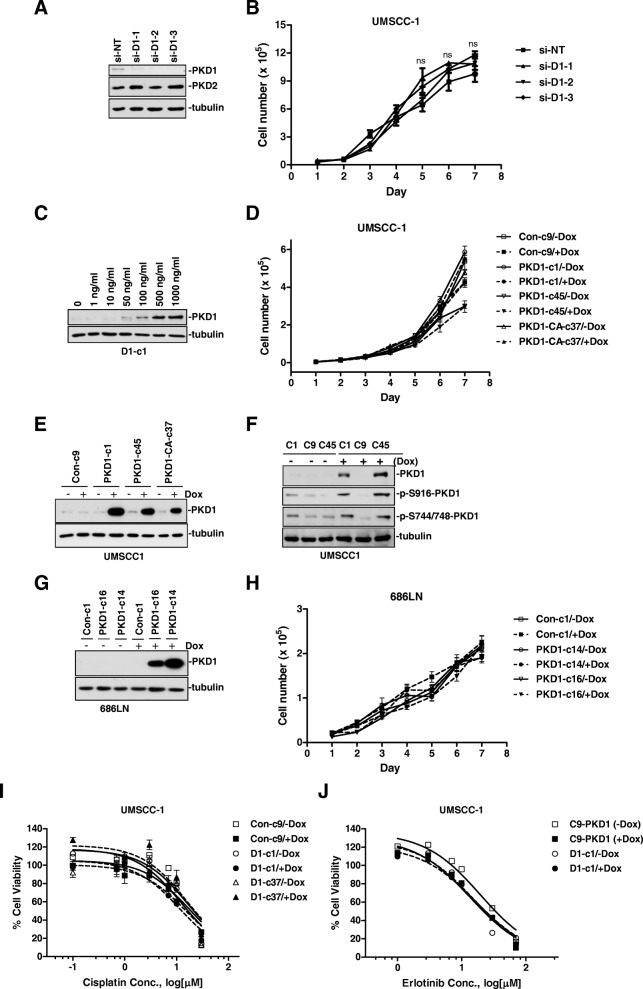
PKD1 was not required for the proliferation and survival of HNSCC cells. **a** and **b** Knockdown of endogenous PKD1 did not affect proliferation of UMSCC-1 cells. Cells were transiently transfected with non-targeting siRNA (si-nt) and three PKD1 siRNAs (si-D1–1, si-D1–2 and si-D1–3). Two days later, transfected cells were replated in triplicates in 24-well plates. Cell growth was determined by counting cell numbers for 7 consecutive days. The experiment was repeated thrice and representative data from one experiment are shown. ns, not significant (*p* ≥ 0.05) by Student’s t test. Knockdown of PKD1 was confirmed by Western blotting. Cell lysates were subjected to immunoblotting for endogenous PKD1–3 and GAPDH (**a**). **c** PKD1 induction by Dox in UMSCC-1 stable PKD1 expression clone (D1-c1). Cells were treated with increasing concentrations of Dox for 48 h before harvesting for Western blotting analysis. **d** Induced PKD1 expression did not alter the proliferation of UMSCC-1 cells. Control (Con-c9), PKD1 (PKD1-c1 and PKD1-c45), and constitutive-active PKD1 (PKD1-CA-c37) expression clones were pre-treated with 500 ng/ml Dox for 2 days. Cells were replated for proliferation assay, as described above. **e** The induction of PKD1 expression by Dox was confirmed by Western blotting. **f** Induction of PKD1 correlated to enhanced basal phosphorylation. Stable inducible clones were treated with Dox at 500 ng/ml for 5 days. The cells were lysed and subjected to Western blotting using p-S738/742-PKD1 and p-S916-PKD1 antibodies. **g** and **h** Induced PKD1 expression did not affect the proliferation of 686LN cells. Control (Con-c1) and PKD1 expression clones (PKD1-c14 and PKD1-c16) were pre-treated with 50 ng/ml Dox for 2 days. Cells were replated for analysis of proliferation by counting cell numbers. The induction of PKD1 expression by Dox was confirmed by Western blotting (**g**). Representative data from one of three independent experiments are shown. ns, not significant (*p* ≥ 0.05) between Dox-treated and -untreated clones. **i** and **j** Induced PKD1 expression did not affect the sensitivity of UMSCC-1 cells to cisplatin and erlotinib. UMSCC-1 cells were seeded in triplicate into 96-well plates and treated with varying concentrations of cisplatin (**i**) and erlotinib (**j**). Media containing fresh cisplatin and erlotinib were replenished every 48 h. The number of viable cells was measured after 72 h using MTT assay. One of two independent experiments is shown. ***P < 0.001

The frequent PKD1 downregualtion in HNSCC tumors and cell lines suggests a potential role of this protein in the pathogenesis of head and neck cancer. To examine this possibility, the functional relevance of PKD1 to tumor-associated biology of HNSCC cells was analyzed systematically in vitro and in vivo. PKD1 has been shown to regulate tumor cell proliferation, survival, migration, and invasion in multiple cancers [1, 20, 25]. Here, the role of PKD1 in HNSCC cell proliferation was first examined by altering PKD1 expression using RNAi or ectopic gene expression approaches. UMSCC-1 cells had been shown to express low but detectable level of PKD1 (Fig. 1a). Using this cell line, endogenous PKD1 was silenced with three siRNAs that target different regions of the PKD1 gene (si-D1–1, si-D1–2, si-D1–3). A non-targeting siRNA (si-NT) was used as the control. The levels of PKD1 knockdown was confirmed by Western blotting analysis (Fig. 4a). Our data indicate that the rate of proliferation of UMSCC-1 cells transfected with si-NT or three different PKD1 siRNAs were not significantly different (Fig. 4b). Thus, knockdown of PKD1 did not significantly affect the proliferation of UMSCC-1 cells.

To determine if enhanced PKD1 expression affect HNSCC cell proliferation, PKD1 was expressed in UMSCC-1 cells using a doxycycline-inducible stable gene expression system. Stable PKD1 clones expressing both Tet-On 3G transactivator protein and PKD1 gene, as well as control clones expressing Tet-On 3G and an empty vector, were established. A total of 7 out of 48 isolated clones were identified as positive PKD1 clones. Additionally, stable clones of a constitutive-active PKD1 harboring a S738/742E mutation in the activation loop (PKD1-CA) were also established. As shown in Fig. 4c, Dox treatment for 48 h concentration-dependently induced PKD1 expression in a stable PKD1 expressor (PKD1-c1). Two positive PKD1 clones (PKD1-c1 and PKD1-c45) and one positive PKD1-CA clone (PKD1-CA-c37) were selected for proliferation assay along with a control clone (Con-c9). There were no significant differences in the proliferation of Con-c9, PKD1-c1, PKD1-c45, and PKD1-CA-c37 clones in the presence or absence of Dox treatment in UMSCC-1 cells (*p* > 0.05) (Fig. 4d). Western blot analysis confirmed that PKD1 was induced upon Dox treatment (Fig. 4e). Additionally, increased PKD1 expression correlated well to enhanced basal phosphorylation of PKD1 at S738/742 and S916, indicating activation of PKD1. The basal PKD1 activity was also examined by immunoprecipitating (IP) PKD1 and directly measuring kinase activity. Our data showed that there was a > 30 fold increase in PKD1 basal activity in PKD1-c1 and PKD1-c45 clones as compared to that in the control PKD1-c9 after 5 days of Dox induction. To ensure that this was not a cell line-specific effect, stable PKD1-inducible clones derived from 686LN were also established and their proliferative properties were evaluated in the presence or absence of Dox treatment. As shown in Fig. 4h, the rate of proliferation was identical for the control (Con-c1) and the PKD1 expressing clones (PKD1-c14, PKD1-c16) with or without Dox treatment. The induction of PKD1 in PKD1-c14, PKD1-c16 clones was confirmed by Western blotting analysis (Fig. 4g). Taken together, these data indicated that the expression and activity of PKD1 were not essential for the proliferation of HNSCC cells.

### PKD1 was not required for the survival of HNSCC cells

PKD1 has been shown to promote cell survival upon activation in many studies [1, 26, 27]. In this study, its role in the survival of HNSCC cells was examined upon the treatment of two chemotherapeutic agents, cisplatin and the EGFR inhibitor erlotinib. UMSCC-1 control (Con-c9), PKD1 (PKD1-c1), and/or PKD1-CA (PKD1-CA-c37) expressors induced with or without Dox were treated with increasing concentrations of cisplatin, followed by the measurement of cell viability by MTT assay. As shown in Fig. 4i, cell survival was not affected by the induced wild-type or constitutive-active PKD1. Similarly, there was no significant difference in the sensitivity of UMSCC-1 control (Con-c9) and PKD1 (PKD1-c1) clones upon treatment of increasing concentrations of erlotinib with or without PKD1 induction by Dox (Fig. 4j). These results demonstrated that overexpression of PKD1 did not affect the sensitivity of UMSCC-1 cells to apoptotic- or cell death-inducing agents, implying that PKD1 is not critical for the survival of HNSCC cells.

### Induced expression of PKD1 potentiated the growth of HNSCC tumor xenografts in nude mice

**Fig. 5:**
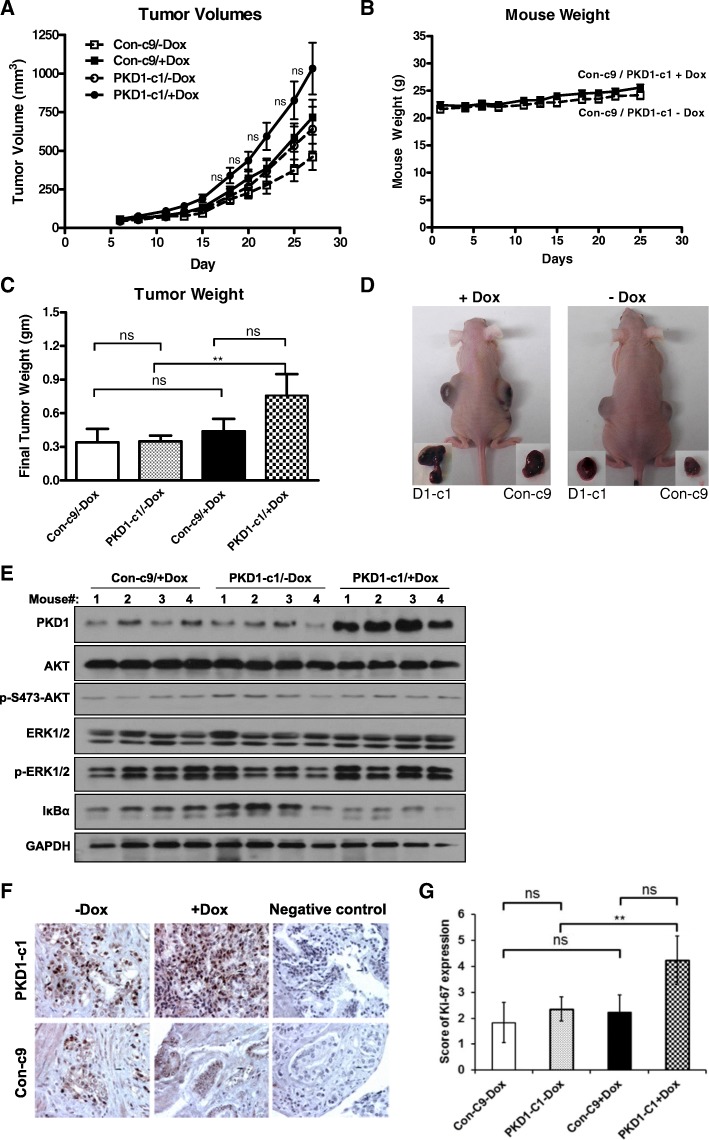
Induced PKD1 promoted the growth of HNSCC tumor xenografts. Control (Con-c9) and PKD1 (PKD1-c1) stable inducible clones were injected subcutaneously in both flanks of female nude mice. Once tumor becomes palpable, mice were randomized into four groups (Con-c9/−Dox, Con-c9/+Dox, PKD1-c1/−Dox, PKD1-c1/+Dox) with Con-c9/+Dox and PKD1-c1/+Dox receiving Dox at 1 mg/ml via drinking water. Tumor volume (**a**) and mouse weight (**b**) were measured every 2–3 days. At the end of the experiment, mice were sacrificed, tumors were dissected and a final tumor weight was recorded for each tumor (**c**). Representative images of mice with their respective excised tumors are shown (**d**). Statistical significance between treatment groups was determined using the Mann-Whitney-Wilcoxon test. ns, *p* ≥ 0.05. Tumor tissues were analyzed by Western blotting for PKD1 and downstream signaling pathways (**e**) and stained for Ki67 (**f**) by IHC and the staining was quantified (**g**). IHC images, × 200. **p < 0.01 by unpaired *t*-test

To evaluate the impact of PKD1 expression on tumor growth in vivo, the growth of tumor xenografts derived from Dox-inducible stable PKD1 overexpressor (PKD1-c1) and the control cells (Con-c9) were examined. PKD1-c1 and Con-c9 were injected subcutaneously in both flanks of female nude mice. Once tumors became palpable, the mice were randomized into four groups with two groups (PKD1-c1/+Dox and Con-c9/+Dox) receiving Dox treatment and two groups (PKD1-c1/−Dox and Con-c9/−Dox) receiving placebo. As shown in Fig. 5a, although the difference in the growth profiles of the tumor xenografts were not statistically significant (*p* > 0.05), the PKD1-c1 clone displayed a trend of faster growth rate, as reflected in the median tumor volume over time, as compared to the control Con-c9. Accordingly, a significant difference in final tumor weight was detected between Dox-treated and untreated PKD1-c1 clones (PKD1-c1/−Dox and PKD1-c1/+Dox) with a higher tumor weight for PKD1-c1/+Dox (**p < 0.01), while there were no statistically significant difference among other groups (*p* > 0.05) (Fig. 5c-d). Notably, no significant signs of toxicity were associated with the Dox treatment or the induction of PKD1, as reflected by the absence of weight loss during entire experiment period (25 days) (Fig. 5b). Tumor tissues were subsequently analyzed by Western blotting for PKD1 expression and downstream signaling activity. As shown in Fig. 5e, PKD1 was induced by Dox treatment in PKD1-c1 mice, while there was no induction of PKD1 in the absence of Dox in PKD1-c1 mice and in the Con-c9 control mice treated with Dox. Dox-treated PKD1-c1 mice also showed elevated p-EKR1/2 and reduced IκBα, indicative of the activation of the MEK/ERK1/2 and the NF-κB signaling pathways. In contrast, the PI3K/Akt signaling pathway was not affected since p-Akt level was not altered. Accordingly, IHC staining showed increased cell proliferation (Ki67) in tumor explants of the Dox-treated PKD1-c1 group as compared with the controls (Fig. 5g). Thus, overexpression of PKD1 promoted the growth of HNSCC tumor xenografts.

### Induced PKD1 expression did not significantly affect the migration and invasion of HNSCC cells

**Fig. 6:**
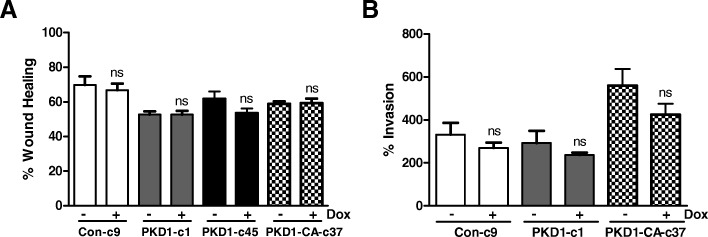
Effects of PKD1 on cell migration and invasion. **a** Wound healing assay. Confluent control (Con-c9), PKD1 (PKD1-c1 and PKD1-c45), and constitutive-active PKD1 (PKD1-CA-c37) expression clones were pre-treated with 500 ng/ml Dox for two days. Monolayers were wounded and imaged immediately (0 h). Wound closure was recorded after 12 h. The width of the wound is the average of 9 determinations per time point. Percent wound healing was calculated at each time point as described in “Materials and Methods”. **b** Matrigel invasion assay. A fixed number of control (Con-c9), PKD1 (PKD1-c1), and constitutive-active PKD1 (PKD1-CA-c37) stable expression cell lines (1.0 × 10^5^/ml) were seeded into the upper control or invasion chamber. After 22 h, non-invading cells were removed and cells that invaded through the matrigel were fixed, stained, and photographed under a microscope. Magnification, 200x. Percent invasion is expressed as the number of cells that invaded through the matrigel matrix relative to the number of cells that migrated through the control insert. Cell number is determined by counting total cell number in random 10 fields. The experiment was repeated thrice and a representative one is shown. ** *P* < 0.01

The role of PKD1in cell migration and invasion has been shown in multiple cancers. Although earlier studies have demonstrated a positive role of PKD1 in regulating cell movement [28–30], later studies provide evidence supporting an opposite effect of PKD1 on cell migration and invasion [8, 10, 31]. In particular, studies conducted in prostate, breast and gastric cancer cells have showed that altered PKD1 expression or activity suppressed tumor cell motility [8, 10, 11]. In this study, the effects of PKD1 on HNSCC cell migration and invasion were examined using wound healing assay and Matrigel invasion assay. PKD1 expression was first induced by treatment with Dox for 48 h in control (Con-c9), PKD1 (PKD1-c1, PKD1-c45), and PKD1-CA (PKD1-CA-c37) expressors. Wound closure was measured 12 h after wounding and % wound healing was calculated. As shown in Fig. 6a, induction of PKD1 or PKD1-CA expression did not significantly affect the rate and extent of wound closure. Similarly, induced expression of PKD1 or PKD1-CA did not significantly alter the invasive properties of these cells as compared to the non-induced controls (Fig. 6b). Thus, PKD1 was not essential for the migration or invasion of HNSCC cells.

### PKD 1 and PKD2 promoted bombesin-induced proliferation of HNSCC cells

**Fig. 7:**
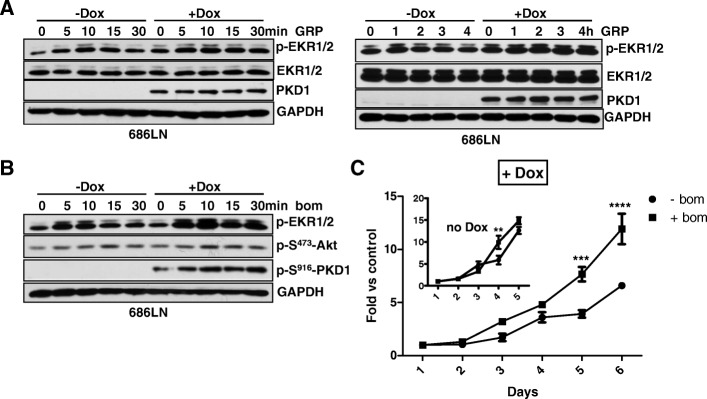
PKD1 overexpression promoted bombesin-stimulated proliferation of HNSCC cells. **a** and **b** Induced PKD1 expression promoted GRP or bombesin -induced ERK1/2 activation in HNSCC cells. Dox-inducible PKD1 overexpressing 686LN cell line was treated with or without Dox at 50 ng/ml for 24 h, followed by GRP or bombesin at 400 nM for the indicated times. The cells were harvested and subjected to Western blotting analysis. Representative images from one of four independent experiments are shown. **c** Induced PKD1 expression promoted bombesin-stimulated proliferation of 686LN cells. PKD1-inducible 686LN cells were pre-treated with 50 ng/ml Dox for 2 days to induce PKD1 expression. Cells with or without induced PKD1 were replated for proliferation assay, followed by treatment with or without bombesin. Cell proliferation in the absence of induced PKD1 (−Dox) treated with or without bombesin was plotted as control (*inset*). Representative data from one of three independent experiments are shown. * *p* < 0.05, ** *P* < 0.01, ***P < 0.001

**Fig. 8:**
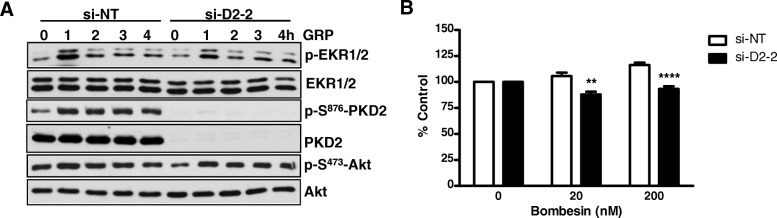
PKD2 knockdown inhibited bombesin-stimulated proliferation of HNSCC cells. **a** PKD2 depletion suppressed GRP-induced ERK1/2 activation. 686LN cells were transfected with a PKD2-targeting siRNA (si-D2–2) and a non-targeting siRNA (si-NT). Two days after transfection, cells were harvested and analyzed by immunoblotting for the indicated proteins. Representative images from one of four independent experiments are shown. **b** Knockdown of PKD2 blocked bombesin-induced proliferation of 686LN cells. 686LN cells transfected with si-NT and si-D2–2 were grown in the absence or presence of bombesin (20, 200 nM) for 3 days. Cell proliferation was measured by MTT assay. Representative data from one of three independent experiments are shown. ** *P* < 0.01, ****P < 0.0001

Bombesin and gastrin-releasing peptide (GRP), the mammalian counterpart of the amphibian tetradecapeptide bombesin [32], exert diverse biological functions in normal and neoplastic tissues through a G protein-coupled receptor known as GRP receptor (GRPR). It has been shown that GRPR mRNA and protein levels are elevated in both HNSCC tumors and adjacent normal mucosa compared with their normal counterparts, and increased GRPR associates with decreased survival in HNSCC patients [33]. Further analysis has identified a GRP-GRPR autocrine loop which contributes to HNSCC growth in vitro and in vivo [33]. The activation of GRPR by GRP and bombesin stimulates the activation of the mitogen-activated protein kinase (MAPK) pathway through transactivating the epidermal growth factor receptor (EGFR) which potentiates HNSCC cell proliferation and invasion [34–36]. Since bombesin-like peptides have been shown to activate PKD and potentiate DNA synthesis in swiss 3T3 cells [37–39], we sought to determine if PKD plays a role in GRP/bombesin-induced mitogenic response in HNSCC. As shown in Fig. 7, in a Dox-inducible PKD1 overexpressing 686LN cell line, bombesin induced potent time-dependent biphasic ERK1/2 activation in the absence of Dox, which peaked at 10 min and 1 h after GRP or bombesin stimulation. In contrast, upon the induction of PKD1, the activation of ERK1/2 was significantly elevated and sustained throughout the treatment period (0–4 h), indicating that overexpression of PKD1 promoted GRP- and bombesin- induced ERK1/2 activity. Functionally, increased PKD1 expression significantly potentiated bombesin-stimulated cell proliferation (Fig. 7b, ‘+Dox’) as compared to the control without PKD1 induction (Fig. 7c, *inset* ‘no Dox’). PKD2 was the predominant isoform expressed in HNSCC cells. It is possible that endogenous PKD2 contributes to GRP- or bombesin- induced ERK1/2 activation and cell proliferation in HNSCC cells. In 686LN cells, GRP induced PKD2 activation, as measured by the increased PKD2 autophosphorylation at S876 (Fig. 8a). Depletion of endogenous PKD2 by siRNA (si-D2–2) significantly blocked GRP-induced p-ERK1/2 signal (most prominently at 1 h), while did not affect GRP-induced Akt phosphorylation. In accordance, despite a limited mitogenic effect was detected in 686LN cells, knockdown of PKD2 completely blocked this effect of bombesin (Fig. 8b), indicating the PKD2 contributes to the bombesin-stimulated mitogenic effect in HNSCC cells. Taken together, these data imply that PKD plays a role in GRP-GRPR induced tumor promotion in HNSCC.

## Discussion

The PKD family has been implicated in a variety of biological processes associated with cancer initiation and progression. PKD1, the most extensively studied PKD isoform, has been shown to be dysregulated in a number of cancer types and plays important roles in tumor cell biology [1, 6, 24, 25]. Interestingly, both tumor suppressive and tumor promoting functions of PKD1 have been reported, which couples to its up- or down-regulation in these tumors, implying a tumor type-specific function of PKD1 in cancer. Specifically, it has been shown that PKD1 is downregulated in invasive human breast tumors as compared to normal breast tissues. Overexpression of constitutively-active PKD1 inhibits the invasion of breast tumor cells, while knockdown of PKD1 confers invasiveness to non-invasive breast cancer cells, an effect that is potentially mediated through negative regulation of MMP expression [8, 40]. In gastric cancer, PKD1 expression is decreased in gastric tumors and cell lines due to PKD1 promoter hypermethylation, and knockdown of PKD1 increased the invasiveness of gastric tumor cell lines [11]. In prostate cancer, PKD1 was downregulated in androgen-independent prostate cancer and increased PKD1 expression blocks cell proliferation and motility [9, 10, 12]. In human osteosarcoma, PKD1 expression in osteosarcoma is significantly lower than that in benign schwannoma samples, and the expression pattern correlated with metastatic potential [41]. Overexpression of PKD1 inhibits osteosarcoma cell proliferation, invasion, and migration and reduced matrix metalloproteinase 2 (MMP2), while knockdown of PKD1 has the opposite effects. Overexpression of PKD1 has also been shown to suppress the growth of osteosarcoma xenografts in vivo [41]. On the other hand, several studies in pancreatic cancer and basal cell carcinoma have demonstrated an opposite role of PKD1 in cancer. PKD1 has been shown to be upregulated both in expression and activity in pancreatic ductal adenocarcinoma as compared to normal pancreatic ducts [27, 42]. The activation of PKD1 promotes pancreatic cancer cell proliferation and increased PKD1 expression contributes to therapy resistance [6, 27, 43–45]. Meanwhile, overexpression of PKD1 significantly promoted DNA synthesis, anchorage-dependent/−independent growth, tumor cell invasion, and angiogenesis in pancreatic cancer cells [44, 45]. In skin cancer, increased PKD1 expression has been demonstrated in basal cell carcinoma lesions as compared to normal epidermis [7]. PKD1 has been associated with pro-proliferative and anti-differentiative phenotypes in epidermis and keratinocytes, implying that PKD promotes hyperproliferative disorders of the skin [7, 46, 47].

HNSCC originates from the mucosal lining of the head and neck regions and accounts for 90% of head and neck cancers. There have not been any studies investigating the role of PKD in head and neck cancer. In this study, we conducted systematically analysis on the expression and function of PKD1 in HNSCC. Our data revealed that the expression of PKD1 was significantly lower in localized HNSCC tumors and metastases, a finding that was further confirmed in patient-paired tumor tissues where PKD1 was downregulated at both mRNA and protein levels in tumors as compared to the normal mucosa. Interestingly, reduced PKD1 expression was re-expressed in only one of five HNSCC cell lines following treatment with histone deacetylase and/or DNA methyltransferase inhibitors. This finding was consistent with the results obtained from cBioPortal analysis of 530 HNSCC tumors from TCGA where low level of DNA methylation on *PRKD1* gene was indicated. Additionally, based on the TCGA data, genetic alteration (LOH or homozygous deletion) only accounts for a fraction of PKD1 mRNA downregulation (~ 13%). Thus, the mechanisms responsible for the downregulation of PKD1 mRNA and protein expression in majority of HNSCC tumors remain to be determined.

Functional analyses of PKD1 using RNAi and stable inducible cell lines revealed that altered PKD1 expression did not significantly affect the proliferation, survival, migration, or invasion of HNSCC cells in the basal state. To ensure that the lack of function was not due to insufficient kinase activity associated with the wild-type protein, a constitutive-active PKD1 mutant was generated and introduced into HNSCC cells and similar results were obtained. Depletion of endogenous PKD1 also did not affect proliferation of UMSCC-1 cells. However, in spite of these findings, some discrepancies were noted when proliferation assays were conducted at different initial seeding density, for example, when the cells were seeded at lower density, such as in Fig. 7c (3000 cells/well), Dox-induced PKD1 appeared to reduce the proliferation of 686LN cells at basal level [comparing Dox-induced and un-induced cells in the absence of bombesin]. However, when the cells were seeded at higher density, such as in Fig. 4h (20,000 cells/well), no difference was observed among cells with or without PKD1 overexpression. Thus, there might be transient and context-dependent growth inhibition by PKD1 at certain cellular context, but this effect is not sustained. Overall, our data consistently showed that either knockdown or overexpression of PKD1 did not significantly alter the proliferation of HNSCC cells in vitro. However, interestingly, induction of PKD1 in vivo by Dox provided a slight growth advantage to the HNSCC tumor xenografts and resulted in a significant increase in final tumor weight in Dox-induced vs the non-induced tumors. This correlated to increased ERK1/2 and NF-κB signaling activity, and enhanced tumor cell proliferation in vivo. Later, we demonstrated that in the presence of a mitogen (bombesin or GRP) that activates PKD, overexpression of PKD1 potentiated the mitogenic effects of bombesin in HNSCC, and depletion of endogenous PKD2, the predominant PKD isoform expressed in HNSCC cells, abolished such effect. At molecular level, overexpression of PKD1 promoted bombein- or GRP-induced ERK1/2 activation, while knockdown of PKD2 reduced EKR1/2 activation. It has been shown that the mitogenic effects of GRP is mediated by the activation of the MEK/EKR1/2 MAPK pathway through transactivating EGFR in HNSCC cells [34]. Our findings imply that PKD1 and PKD2 may contribute to the mitogenic effect of GRP and bombesin by facilitating the activation of ERK1/2. This is a novel interesting finding that unlike other reports showing a significant functional role that associates with altered PKD1 expression in different tumors, our data indicate that PKD1 has limited functional impact in the proliferation, survival, migration, and invasion of HNSCC cells at the basal state, despite frequent downregulation of *PRKD1* transcript and protein expression in HNSCC tumors and cell lines. Importantly, under in vivo condition or in the presence of an activating mitogen such as GRP or bombesin, PKD1 and PKD2 act to promote tumor cell proliferation. Our results and others suggest that the biological functions of PKD may be cancer type- and cell context- dependent. Perhaps the function of PKD is contingent on another yet unknown protein/pathway in certain cancer types. Meanwhile, high expression of another PKD isoform, such as PKD2, may substitute the need for other PKD isoforms. In a recent report in gastric cancer where low PKD1 and high PKD2 expression were detected in poorly differentiated adenocarcinoma, overexpression of PKD1 had no/minimal effects on tumor cell survival and proliferation, which is consistent with our findings [26]. In HNSCC, PKD2 was the predominant PKD isoform expressed in HNSCC cells. PKD2 mRNA was upregulated in seven out of ten tumors vs normal in patient-paired HNSCC tissue specimens. Thus, it is possible that PKD2 plays a predominant role in the growth, survival, and motility of HNSCC cells, and these functions have compensated the loss of PKD1 in tumors, our data from PKD2-knockdown cells support this claim.

## Conclusions

Our study has demonstrated significant downregulation of PKD1 in HNSCC tumors and metastases. However, despite its reduced expression,, PKD1 remains a pro-proliferative signaling protein in HNSCC cells upon activation by GRP or bombesin, implying an important role of PKD1 in GRP/bombesin-induced oncogenesis in HNSCC. Our study also highlights the complexity of PKD-associated biology and the need for caution when analyzing biological functions of frequently downregulated genes in certain cancers.

## Additional file

Additional file 1: **Figure S1** Levels of *PRKD1* gene transcript and DNA methylation in HNSCC (TCGA). **A.** Provisional data on CNAs of the *PRKD1* gene in HNSCC (TCGA) were adapted from cBioPortal. **B.** *PRKD1* methylation as a function of mRNA expression. Provisional data on *PRKD1* methylation in HNSCC (TCGA) adapted from cBioPortal. **Figure S2** DNA methyltransferase and HDAC inhibitors did not affect PKD1 expression in HNSCC cell lines. UPCI14B, OSC19, and Cal33 cells were treated with SAHA and 5-aza-dC alone or in combination for 48 h. Cells were lysed and subjected to Western blotting for PKD1 expression levels. Het-1A was used as a control. GAPDH was blotted as a loading control. Representative data from one of three independent experiments are shown. (PPT 3567 kb)

## Abbreviations

5-aza-dC: 5-Aza-2′-deoxycytidine
CaMK: Ca^2+^/calmodulin-dependent protein kinase
Dox: Doxycycline
EGFR: Epidermal growth factor receptor
GPCRs: G protein-coupled receptors
HNSCC: Head and neck squamous cell carcinoma
IHC: Immunohistochemistry
MAPK: Mitogen-activated protein kinase
MMP2: Matrix metalloproteinase 2
PKD: Protein kinase D
