# Supplementary material for: Analysis of oncogenic activities of protein kinase D1 in head and neck squamous cell carcinoma
Source: BMC Cancer. 2018 Nov 12;18:1107. doi: 10.1186/s12885-018-4965-6 (PMC6233608; doi:10.1186/s12885-018-4965-6)
Supplement: Supplementary file 1 — Figure S1 Levels of PRKD1 gene transcript and DNA methylation in HNSCC (TCGA). A. Provisional data on CNAs of the PRKD1 gene in HNSCC (TCGA) were adapted from cBioPortal. B. PRKD1 methylation as a function of mRNA expression. Provisional data on PRKD1 methylation in HNSCC (TCGA) adapted from cBioPortal. Figure S2 DNA methyltransferase and HDAC inhibitors did not affect PKD1 expression in HNSCC cell lines. UPCI14B, OSC19, and Cal33 cells were treated with SAHA and 5-aza-dC alone or in combination for 48 h. Cells were lysed and subjected to Western blotting for PKD1 expression levels. Het-1A was used as a control. GAPDH was blotted as a loading control. Representative data from one of three independent experiments are shown. (PPT 3567 kb) [file 12885_2018_4965_MOESM1_ESM.ppt]

## Slide 1
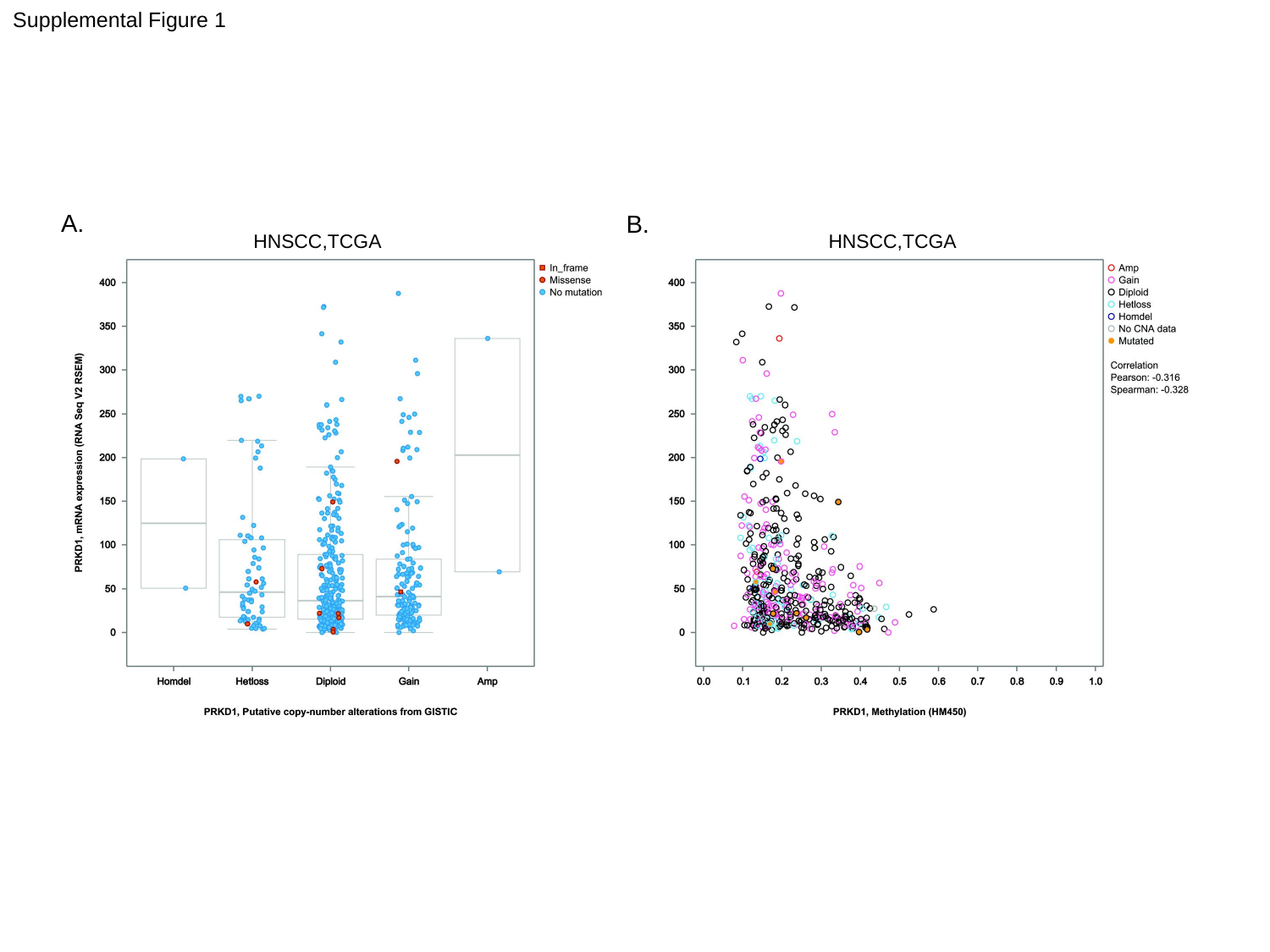

Supplemental Figure 1
A.
B.
HNSCC,TCGA
HNSCC,TCGA

## Slide 2
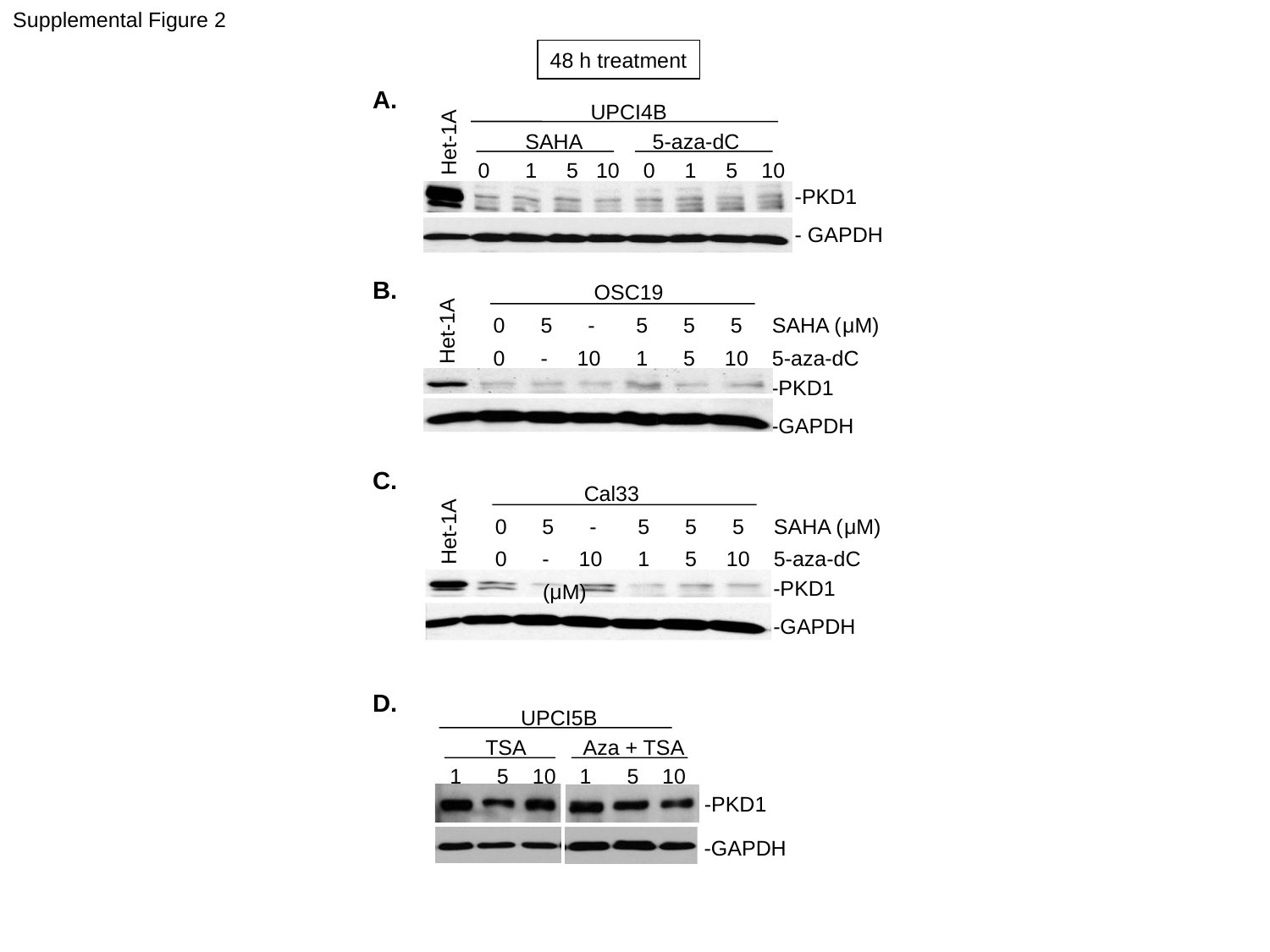

Supplemental Figure 2
48 h treatment
A.
 UPCI4B
 SAHA 5-aza-dC
0 1 5 10 0 1 5 10
Het-1A
-PKD1
- GAPDH
 OSC19
0 5 - 5 5 5 SAHA (μM)
0 - 10 1 5 10 5-aza-dC (μM)
B.
Het-1A
-PKD1
-GAPDH
C.
 Cal33
0 5 - 5 5 5 SAHA (μM)
0 - 10 1 5 10 5-aza-dC (μM)
Het-1A
-PKD1
-GAPDH
D.
 UPCI5B
 TSA Aza + TSA
1 5 10 1 5 10
-PKD1
-GAPDH
